# Cholera, Its Prevention and Treatment

**Published:** 1884-08

**Authors:** 


"CHOLERA, ITS PREVENTION AND TREATMENT," by D. N. Ray, M. D., Calcutta, with Introductory by T. F. Allen, M. D., New York, will be published August 1st, by A. L. Chatterton Publishing Co. Price, $1.00. Cloth.

